# Examining the involvement of Slx5 in the apoptotic response to chronic activation of the spindle assembly checkpoint

**DOI:** 10.3906/biy-1812-46

**Published:** 2019-06-13

**Authors:** Pınar Buket ATALAY, Elif Ergin ÇAVUŞOĞLU, Öykü AŞCI, Duygu AYGÜNEŞ

**Affiliations:** 1 Department of Medical Biology and Genetics, Faculty of Medicine, Maltepe University, İstanbul, Turkey; 2 Department of Clinical Embryology, Maltepe University Graduate School of Health Sciences, Maltepe University, İstanbul, Turkey; 3 Department of Medical Biology, Faculty of Medicine, Ege University, İzmir, Turkey

**Keywords:** Saccharomyces cerevisiae, spindle assembly checkpoint, cancer, prolonged mitotic arrest, apoptosis

## Abstract

Microtubule-targeting agents represent one of the most successful groups of anticancer drugs used in cancer therapy today. These drugs induce a prolonged mitotic arrest through chronic spindle assembly checkpoint (SAC) activation. Apoptosis, an outcome of the prolonged mitotic arrest, is the main mechanism by which these anticancer drugs kill cancer cells. However, not much is known about the mechanism that directs chronic SAC activation to apoptosis among other possible outcomes. The aim of this study is to investigate whether Slx5, a sumo-targeted ubiquitin E3 ligase, is involved in directing chronic SAC activation to apoptosis. We show that chronic SAC activation triggered by a 10-h nocodazole incubation leads to a prolonged mitotic arrest in the *slx5*Δ strain similar to wild type (WT). However, the proportion of cells displaying apoptotic features such as nuclear fragmentation, DNA fragmentation, and reactive oxygen species (ROS) production were increased more in the WT strain during the chronic SAC activation compared to *slx5Δ*, indicating that Slx5 may be involved in the chronic SAC-activation-apoptosis relation. We also showed that the possible role of Slx5 in the chronic SAC activation-apoptosis association was not through ubiquitin dependent degradation of 3 apoptosis-related and sumoylated candidate proteins.

## 1. Introduction

The spindle assembly checkpoint (SAC) is a conserved major cell cycle checkpoint, which ensures high fidelity of chromosome segregation in mitosis. SAC monitors kinetochore-microtubule attachments. In the presence of improper attachment(s), SAC activation leads to a cell cycle arrest in metaphase until the attachment error is corrected before the cell proceeds to anaphase, thus prevents aneuploidy in daughter cells (Lara-Gonzalez et al., 2012). Since SAC activation prevents cell proliferation by inducing a mitotic arrest in response to improper kinetochore-microtubule attachments, microtubules represent one of the most successful chemotherapeutic targets in clinic today. Two groups of anticancer drugs, taxanes (paclitaxel/taxol, docetaxel) and vinca alkaloids (vinblastine, vincristine) that are successfully used in the treatment of several types of cancer in clinic, prevent cancer proliferation by targeting microtubules (Hadfield et al., 2003; Marques et al., 2015). Both groups of drugs disrupt spindle microtubules, thus prevent proper attachments. Lack of proper attachments due to the drug treatment induces chronic SAC activation, which leads to a prolonged mitotic arrest (Gascoigne and Taylor, 2009). Since mitosis is considered the most vulnerable cell cycle phase to various environmental factors including radiation (Stobbe et al., 2002) and chemicals (Hughes, 1950), mitotic arrest induction (increasing the time cells spend in mitosis) is favorable for cancer treatment. SAC-activating antimitotic drugs have been reported to induce a prolonged mitotic arrest in all cancer cell lines tested (Huang et al., 2010). Different cell fates have been identified following the prolonged mitotic arrest. Among these, “apoptosis” (programmed cell death) has been widely accepted as one of the main mechanisms through which anticancer drugs kill cancer cells (Atalay and Aydın, 2017). Recent studies revealed the link between chronic SAC activation and apoptosis by showing that Mcl1, a human antiapoptotic protein, is degraded by a Cdk1/cyclin B phosphorylation and APC/C dependent mechanism (Harley et al., 2010; Wertz et al., 2010). A DNA damage response mechanism activated the damage accumulated in mitotically arrested cells (Imreh et al., 2011; Hayashi et al., 2012) has also been shown to induce apoptosis in these cells (Hain et al., 2016). However, the nature of the signal produced as a result of chronic SAC activation and its relation to the apoptotic mechanism is largely unknown.Apoptosis, a highly regulated mode of programmed cell death, is a vital component of normal development and aging, as it is essential for maintaining tissue homeostasis by balancing cell death and cell division. Although different apoptotic pathways require specific signals to be triggered, they all converge on the same execution pathway. Activation of the execution pathway leads to the apoptosis specific characteristics such as caspase activation, DNA fragmentation, and membrane blebbing (Elmore, 2007). Although apoptosis has been considered a metazoan programmed cell death mechanism, *Saccharomyces cerevisiae *has also been demonstrated to undergo cell death with apoptotic features over two decades ago (Madeo et al., 1997). Since then, several studies reported that yeast cells undergo apoptosis in response to various stress conditions including acetic acid, hydrogen peroxide, high salt or sugar (Madeo et al., 2004). Chronic SAC activation-induced prolonged mitotic arrest also triggers apoptosis in *S. cerevisiae *(Endo et al., 2010). Slx5 (the yeast orthologue of human RNF4) is a subunit of the budding yeast heterodimeric Slx5/Slx8 sumo-targeted ubiquitin ligase complex. Sumoylation is a reversible posttranslational modification, which involves covalent attachment of small ubiquitin-related modifier (sumo) to the substrate lysine residues on target proteins (Flotho and Melchior, 2013). Several proteins involved in many key cellular processes including DNA damage repair, cell cycle, transcription, and chromosome segregation are known to be sumoylated. Sumoylation is important for response to various stress conditions such as heat shock, oxidative stress, DNA damage, and ethanol stress (Enserink, 2015), and has been shown to be associated with apoptosis in different cell lines (Sudharsan ve Azuma, 2012; Jin et al., 2017; Xiu et al., 2018), as well as *S.cerevisiae* (Owsianowski et al., 2008). Ubiquitination of the sumoylated proteins is critical for the turnover of the sumoylated proteins, because sumo-targeted ubiquitin ligases can specifically induce degradation of sumoylated proteins (Geoffroy and Hay, 2009). A large number of proteins are known to be sumoylated in *S.cerevisiae* as well: A proteomic analysis of sumoylation revealed a total of 251 sumo-conjugated proteins in yeast (Denison et al., 2005). In this study, we investigated whether Slx5 is involved in the apoptotic response induced by chronic SAC activation. Our data revealed that chronic SAC activation induces a prolonged mitotic arrest in *slx5*Δ cells similar to wild type cells. However, the percentage of *slx5*Δ cells displaying apoptotic features of nuclear fragmentation, DNA fragmentation and intracellular ROS (reactive oxygen species) production following the chronic SAC activation was lower compared to wild type cells, suggesting that Slx5 may be involved in the chronic SAC activation-apoptosis relation. We also showed that the possible role of Slx5 in chronic SAC activation-apoptosis relation is not through ubiquitin dependent degradation of 3 apoptosis-related and sumoylated candidate proteins. 

## 2. Materials and methods

### 2.1. Strains and growth conditions

All *Saccharomyces cerevisiae* strains (WT,* slx5Δ *and TAP-tagged strains) used in this study were in the BY4741 background (*MATa* *his3Δ1 leu2Δ0 met15Δ0 ura3Δ0)*. WT TAP-tagged strains were a kind gift from Dr. Daniel J. Burke (North Carolina State University, College of Sciences, Department of Biological Sciences). *slx5Δ* was deleted in each WT TAP-tagged strain by one-step gene replacement. s*lx5Δ::KANMX *was amplified by PCR and transformed into the WT TAP-tagged strains by lithium-acetate transformation method as described previously (Gietz and Schiestl, 2007). All strains were maintained on YPD agar plates containing 1% (wt/vol) yeast extract, 2% (wt/vol) peptone, 2% (wt/vol) glucose, and 2% (wt/vol) agar and cultured in liquid YPD medium (2% wt/vol) glucose, 1% (wt/vol) yeast extract and 2% (wt/vol) peptone) shaking (175 rpm) at 30°C.

### 2.2. Chronic SAC activation

In order to induce chronic SAC activation, WT and *slx5Δ* strains were grown to early mid-logarithmic phase (OD600 = ~0.3) and arrested in G1 with α-factor (200 µM) in acidic medium (pH = 3.4) at 30 °C for 2 h. G1 synchronization was considered successful when more than 90% of the cells were observed to be unbudded under the light microscope. G1-arrested cells were removed from α-factor containing medium by washing 3 times with dH2O and released into nocodazole containing medium (15 µg/ml) (0 h) and incubated for 10 h (10 h). Nocodazole (15 µg/ml) was readded 5 h after their release into the nocodazole-containing medium. Samples collected from both strains at every 2 h (0 h, 2 h, 4 h, 6 h, 8 h, 10 h) were examined for SAC activation, apoptotic features, and concentrations of TAP-tagged candidate proteins.

### 2.3. Western blot analysis

Protein extracts of samples taken from WT and *slx5Δ* cultures at every 2 h for 10 h were prepared by the NaOH method as described previously (Kushnirov, 2000). The whole cell extracts were separated on 10% SDS-containing polyacrylamide gel and transferred onto immobilon PVDF membrane (Millipore). The membranes, blocked in 5% dry fat-free milk in 1XPBS (phosphate-buffered saline) for at least 1 h at room temperature, were probed with anti-Pds1 (Santa Cruz), anti-Clb2 (Santa Cruz) for mitotic arrest detection, and with anti-TAP (Invitrogen) for the detection of TAP-tagged proteins. Anti-Pgk1 (Abcam) was used for the detection of the loading control (Pgk1). Following the primary antibody incubations overnight at 4°C, membranes were washed three times with 1XPBS and incubated with the appropriate HRP-conjugated secondary antibodies in 5% dry fat-free milk in 1XPBS for 1 h at room temperature. Following three washes with 1XPBS, the blots were stained with ECL (WesternBright Sirius HRP substrate, Advansta), exposed to MXBE blue film (Carestream) (for 5 s to 2 min, depending on the detected protein) and manually developed. Pictures of the films were taken on a light box. Pds1 and Clb2 levels in WT and* slx5Δ* were analyzed in at least two independent experiments by western blot. Pds1 and Clb2 band intensities were quantified using the ImageJ software (Scion Corp, Bethesda, Maryland, USA). The double band observed for Clb2 may be due to the detection of two splice variants of Clb2 by the anti-Clb2 antibody. 

### 2.4. Flow cytometry analysis

Cells were fixed with 70% ethanol at room temperature and kept at 4°C until flow cytometry analysis. Prior to the analysis, the cells were rehydrated in water and treated with pepsin for 30 min at 37°C. Pepsin was removed from the samples by washing with dH2O 3 three times. The cells were then treated with RNAse at 37°C overnight. Cells were stained with sybr green for 5 min in the dark and sonicated prior to the analysis. Flow cytometry analyses were performed on AccuriTM C6 flow cytometer (BD biosciences). A total of 40.000 cells at each time point were evaluated for DNA content.

### 2.5. DAPI (4′, 6-diamidino-2-phenylindole) staining

Samples collected at each time point were fixed with 3.7% (v/v) formaldehyde for 1.5 h at room temperature. Following the fixation, samples were washed twice with dH2O, resuspended in dH2O, and kept at 4°C until DAPI staining. For DAPI staining, samples were centrifuged and fixed with 70% (v/v) ethanol for 30 min at room temperature, washed once with dH2O, and resuspended in dH2O. Five μL of the samples were loaded onto microscope slides and stained with DAPI (4′, 6-diamidino-2-phenylindole) (2 μg/ml) in Vectashield mounting medium (Vector, Burlingame, CA). At least 200 cells for each time point were evaluated for nuclear fragmentation under a fluorescence microscope (Leica DM1000 LED, Leica Microsystems, Germany) and categorized as “fragmented” or “unfragmented”. The experiment was conducted three times for each strain and the average of the “percent fragmented nucleus” was reported with standard error of the mean.

### 2.6. TUNEL (Terminal deoxynucleotidyl transferase dUTP nick-end labeling) assay

DNA fragmentation was analyzed by TUNEL assay as described previously (Madeo et al., 1997) with some modifications. Briefly, samples were fixed with 3.7% (vol/vol) formaldehyde for 1-1.5 h at room temperature. Following the fixation, cells were washed with (PBS) three times, resuspended in dH2O, and maintained at 4°C until the TUNEL staining. For TUNEL staining, yeast cell walls were digested with 24 μg/ml Zymolyase 100T (MP Biomedicals) at 37°C for 1 h. Ten mL of the cell suspension was applied onto a polylysine-coated microscope slide and allowed to dry for 30 min at 37°C. After rinsed with PBS, the slides were permeabilized with 0.1% (vol/vol) Triton X-100 and 0.1% (wt/wt) sodium citrate for 2 min on ice, rinsed twice with PBS, and incubated with 10 μL TUNEL staining reaction mixture (in situ cell death detection kit, fluorescein, Roche diagnostics) at 37°C for 1 h in the dark and the slides were washed three times with PBS. The slides were mounted with a drop of Kaiser’s glycerol gelatin (Merck) and approximately 200 cells at each time point were examined for TUNEL staining under a fluorescence microscope (Leica DM1000 LED, Leica Microsystems, Germany) and scored as “TUNEL positive” and “TUNEL negative”. The average “percent TUNEL positive cells” of three independent experiments was reported for each strain with standard error of the mean. 

### 2.7. Detection of reactive oxygen species (ROS)

Intracellular ROS levels in each strain were examined as described previously (Madeo et al., 1999). Briefly, 200 μL samples taken at every time point were resuspended in fresh YPD and incubated with 10 μg/ml 2′, 7′-dichlorodihydrofluorescein diacetate (H2DCFDA; molecular probes) at 30°C for 40 min. Following the incubation, 5 μL of each sample was spotted onto microscope slides and observed under a fluorescence microscope (Leica DM1000 LED, Leica Microsystems, Germany, excitation 495 nm and emission 525 nm). At least 200 cells for each time point were examined for DCF fluorescence. The experiment was conducted in triplicate for each strain and the average “percent DCF positive” cells were reported with standard error of the mean.

### 2.8. Statistical analyses 

Each experiment that was analyzed statistically was repeated three times. Data represent standard error of the mean. Difference between the 0th and the 10th hour of the nocodazole incubation was examined using student’s t-test, the difference considered statistically significant when P < 0.05.

## 3. Results

### 3.1. Detection of the chronic SAC activation-dependent prolonged mitotic arrest

**Figure 1:**
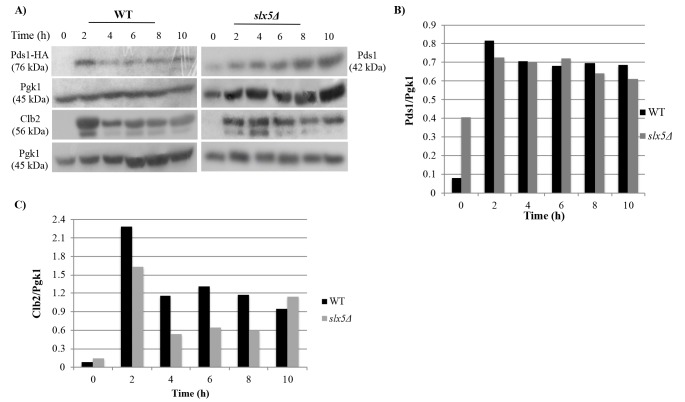
Western blot analysis of SAC-dependent prolonged mitotic arrest. G1 synchronized cells were released into nocodazole (0 h) and incubated for 10 h. (A) Pds1 and Clb2 levels were analyzed by western blot, and Pgk1 was used as the loading control. Relative band intensities were determined using the ImageJ software. (B) Relative intensities of Pds1 bands were calculated by dividing the intensity of the Pds1 band by the intensity of the Pgk1 band at the same time point. (C) Relative intensities of Clb2 bands were calculated by dividing the intensity of the Clb2 band by the intensity of the Pgk1 band at the same time point.

**Figure 2:**
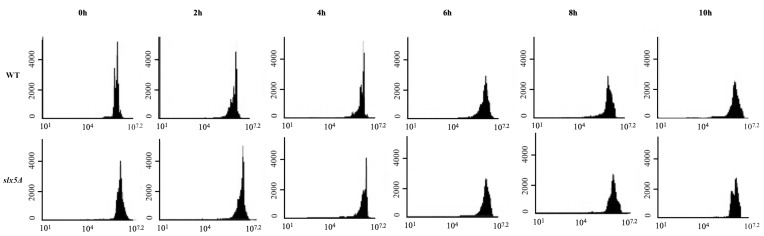
Flow cytometry analysis of SAC-dependent prolonged mitotic arrest. Synchronized WT and slx5Δ cells were released into the nocodazole-containing media (0 h) and incubated for 10 h. DNA content of the samples were analyzed by flow cytometry. A total of 40.000 cells were evaluated at each time point.

In order to investigate whether *slx5*Δ cells display a SAC-dependent prolonged mitotic arrest in response to long-term treatment with nocodazole, an antimicrotubule drug,*slx5Δ* and WT cells at early midlogarithmic phase were synchronized in G1 with and released into nocodazole containing medium for 10 h to trigger chronic SAC activation. Samples collected from both strains at every 2 h (0 h, 2 h, 4 h, 6 h, 8 h, 10 h) were examined for Pds1 and Clb2 concentrations by western blot (Figure  1) and for DNA content by flow cytometry (Figure  2) analyses. Degradation of Pds1, the anaphase inhibitor, is a widely accepted biochemical marker for metaphase-to-anaphase transition in *S. cerevisiae*. Pds1 levels reach their maximum in metaphase and decrease as the cells proceed into anaphase in cycling cells. SAC activation leads to stabilization of Pds1 levels due to metaphase-arrest induction (Yamamoto et al., 1996). Western blot analyses of Pds1 levels in WT and *slx5*Δ cells revealed that in *slx5*Δ cells, Pds1 was expressed 2 h after the α-factor release and remained stable during the course of the experiment similar to WT cells (Figure  1A, 1B), suggesting that *slx5*Δ cells arrest in metaphase in response to prolonged nocodazole treatment. Another biochemical marker for mitosis is the expression of Clb2, which is the yeast mitotic cyclin. Degradation of Clb2 is required for the cells to exit from mitosis (Wäsch and Cross, 2002). Consistent with the Pds1 results, Clb2 was expressed 2 h after the α-factor release and was not degraded even in the last time point (10th h) of the experiment in *slx5*Δ cells similar to WT cells (Figure  1A, 1C), indicating that *slx5*Δ cells stay in mitosis during the course of the experiment. We also investigated mitotic arrest through examining the DNA content of single cells by flow cytometry. In support of the western blot results, flow cytometry analysis also showed that both *slx5*Δ and WT cells enter mitosis 2 h after the α-factor release with a 2C DNA content and maintain the 2C content during the course of the experiment (Figure  2). These data together indicate that *slx5*Δ cells arrest in mitosis in response to prolonged exposure to nocodazole, revealing that they have an intact SAC activity.

### 3.2. Examining apoptotic features during the prolonged mitotic arrest 

**Figure 3:**
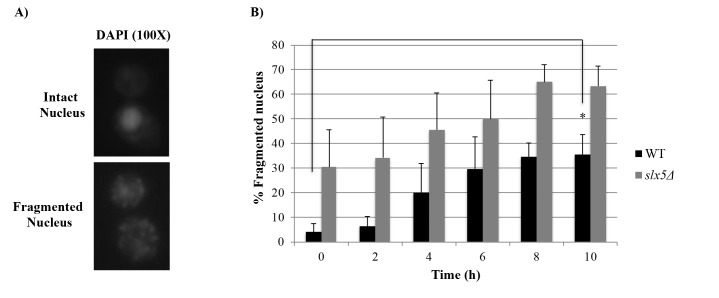
Nuclear morphology by DAPI staining. (A) Nuclei of DAPI stained cells were categorized as “intact” or “fragmented” under the fluorescent microscope during the prolonged mitotic arrest. (B) At least 200 cells were examined at each time point and the ratio of cells with fragmented nuclei was graphed. Data represent the standard error of the mean of three independent experiments. *: P < 0.05.

**Figure 4:**
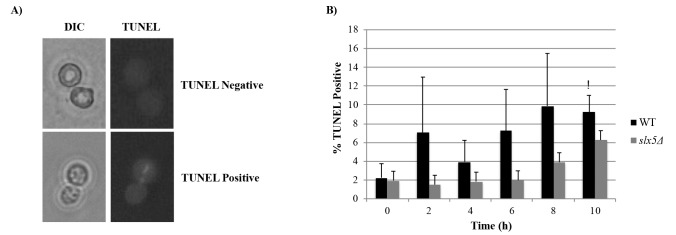
DNA fragmentation by TUNEL staining. (A) TUNEL stained cells were categorized as “TUNEL positive” or “TUNEL negative” under the fluorescent microscope. (B) For both WT and slx5Δ strains, at least 200 cells were evaluated for TUNEL staining and the ratio of TUNEL positive cells were graphed. Data represent the standard error of the mean of three independent experiments. *: P < 0.05.

**Figure 5:**
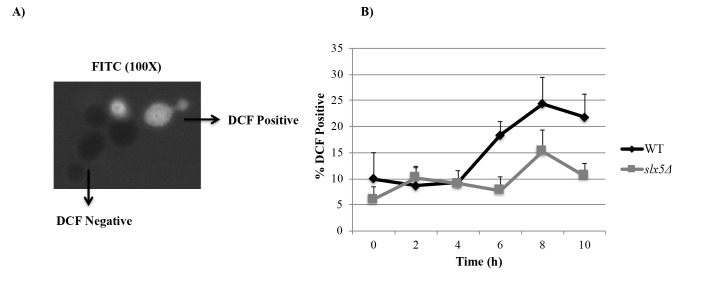
ROS detection. (A) Samples were examined for DCF signal under the fluorescence microscope and categorized as “DCF positive” or “DCF negative”. (B) At least 200 cells were evaluated for each strain at each time point and percentage of cells with fluorescent DCF signal was graphed. Data represent the standard error of the mean of three independent experiments.

WT budding yeast cells have been previously reported to undergo mitotic cell death with apoptotic features in response to prolonged (10-h) nocodazole treatment (Endo et al., 2010). After we observed that 10-h nocodazole treatment leads to a prolonged arrest in* slx5*Δ cells as well, we investigated whether Slx5 is involved in the apoptotic response to the prolonged nocodazole treatment by comparing the proportion of WT and *slx5*Δ cells displaying apoptotic features during the prolonged nocodazole treatment. For this purpose, α-factor-synchronized WT and *slx5*Δ cells were incubated in nocodazole containing medium for 10 h. Samples taken from both cultures (0 h, 2 h, 4 h, 6 h, 8 h, 10 h) were examined for some apoptotic features.Nuclear fragmentation is a morphology of apoptosis also in yeast (Madeo et al., 1997). Samples collected from WT and *slx5*Δ cultures were investigated for their nuclear morphologies by DAPI staining and categorized as “intact” or “fragmented” under the fluorescence microscope (Figure  3A). We observed that percentage of cell with a fragmented nucleus (%Fragmented nucleus) in *slx5Δ *was higher compared to that of WT at each time point: %Fragmented nucleus in *slx5Δ *and WT strains at the 10th h of nocodazole incubation were 63.5% and %34.5, respectively. However, is noteworthy that the ratio of cells with a fragmented nucleus in the *slx5Δ *culture at the 0th h of the nocodazole treatment (30.5%) was also higher compared to WT at the same time point (4.1%) (Figure  3B). When we compared %Fragmented nucleus at the 10th h with the 0th h, we observed that %Fragmented nucleus in WT cells at the 10th hour (35.4%) was significantly higher compared to 0th hour (4.1%) (8.6-fold increase, P < 0.05). However, in the *slx5Δ* strain, there was no significant difference in the percentage of cells with fragmented nucleus between the 0th (30.5%) and 10th h (63.5%) of nocodazole treatment (2.1-fold increase, P > 0.05) (Figure  3B).Fragmentation of DNA into oligonucleosomal size fragments is a typical morphology of yeast apoptosis as it is in mammals (Ribeiro et al., 2006). DNA fragmentation in WT and *slx5*Δ cells during the prolonged mitotic arrest was examined using the TUNEL (terminal deoxynucleotidyl transferase dUTP nick-end labeling) assay, which is based on labeling free 3’-OH ends of DNA fragments with fluorescent-conjugated dUTP, and the cells were categorized as “TUNEL positive” or “TUNEL negative” under the fluorescent microscope (Figure  4A). Similar to the DAPI results, %TUNEL Positive cells in WT cells at the 10th h (9.2%) was significantly higher compared to the 0th h (2.2%) (4.1-fold increase, P < 0.05), whereas in *slx5Δ *cells %TUNEL positive cells at the 10th h (6.3%) was not significantly different from the 0th h (1.9%) (3.2-fold increase, P > 0.05) (Figure  4B).Reactive oxygen species (ROS) are important cell death regulators that are known to be associated with apoptotic pathways in *S.cerevisiae* as well as in complex eukaryotes (Carmona-Gutierrez et al., 2010). ROS accumulation in cells is known to be important in inducing apoptosis (Perrone et al., 2008). Therefore, we examined intracellular ROS production in response to prolonged nocodazole treatment in *slx5Δ* cells by the H2DCFDA assay (Figure  5). The assay is based on the conversion of H2DCFDA to a strong fluorescent DCF upon oxidation by ROS in the cells. Cells were categorized as “DCF negative” or “DCF positive” under the fluorescent microscope (Figure  5A) and %DCF positive cells were plotted (Figure  5B). Although %DCF positive cells in WT and *slx5Δ *strains were not statistically different at any time point during the experiment, %DCF positive cells were increased more in the WT strain at the 10th h of the nocodazole treatment compared to the 0th h (2.2-fold) relative to that of *slx5Δ* strain (1.7-fold) (Figure  5B).

### 3.3. Investigating the role of Slx5 in the chronic SAC activation-apoptosis relation

**Figure 6:**
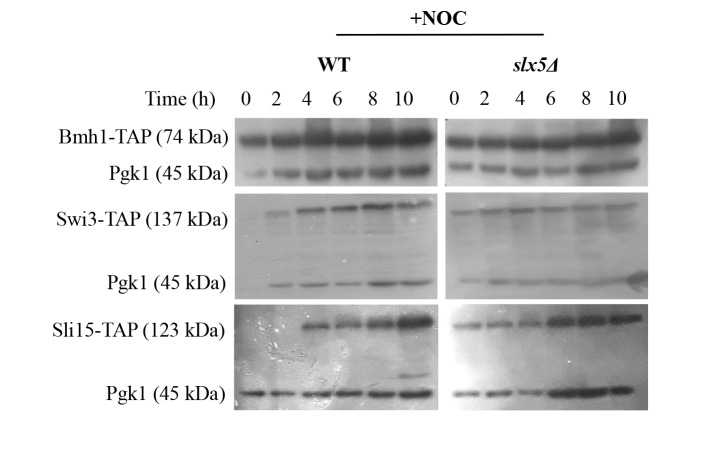
Western blot analyses of TAP-tagged proteins. G1-synchronized WT and slx5Δ cells were released into nocodazole containing (+NOC) media and incubated for 10 h. Concentrations of the TAP-tagged proteins were analyzed by western blot, and Pgk1 was used as the loading control.

Our data indicate that Slx5 may be involved in the apoptotic response to prolonged mitotic arrest triggered by chronic SAC activation. Therefore, we finally tested whether the possible role of Slx5 in the chronic SAC activation-apoptosis relation is through its sumo-targeted E3 ubiquitin ligase activity. For this purpose, we examined and compared the concentrations of candidate proteins in WT and *slx5*Δ strains under chronic SAC activating (+NOC) conditions for 10 h by western blot analysis (Figure  6). Bmh1, Swi3, and Sli15 were selected as “candidates”, because these proteins are thought to be apoptosis-related by their own or their binding partner’s mutant phenotypes and were also shown to be sumoylated in yeast (Denison et al., 2005). We did not detect any dramatic differences in the concentrations of the candidate proteins between the two strains at any time point throughout the experiment (Figure  6). These data suggest that the role of Slx5 in the chronic SAC activation-apoptosis relation is not through ubiquitin dependent degradation of the candidate proteins. 

## 4. Discussion 

Antimitotics, one of the most effective group of drugs used in cancer therapy today (van Vuuren et al., 2015), trigger chronic SAC activation, which led to a prolonged mitotic arrest in all cancer cell lines tested (Huang et al., 2010). Cells can undergo different fates following the prolonged arrest. Among these fates, apoptosis is a major mechanism through which antimitotic drugs kill cancer cells (Mukhtar et al., 2014). Another outcome of the prolonged mitotic arrest, slippage, is an important factor that significantly reduces the effectiveness of chemotherapy (Matson and Stukenberg, 2011). Therefore, to be able to direct chronic SAC activation dependent prolonged mitotic arrest to the apoptosis outcome is important to increase chemotherapy effectiveness. Although induction of apoptosis following the prolonged mitotic arrest triggered by antimitotics is a desirable outcome of cancer therapy, the nature of the signal produced during the mitotic arrest and how it is related to the apoptotic mechanism is largely unknown. Here we investigated the role of Slx5, a sumo-targeted E3 ubiquitin ligase, in the chronic SAC activation dependent prolonged mitotic arrest-apoptosis relation. Western blot analysis of Pds1 and Clb2 levels as well as flow cytometry analysis of the DNA content during the experiment revealed that chronic SAC activation triggered by a 10-h nocodazole incubation induces a prolonged mitotic arrest in both WT and *slx5*Δ cells (Figure  1), suggesting that Slx5 is not required for chronic SAC activation. Our data on WT cells support the previous study, which reported that WT budding yeast cells arrest in mitosis in response to chronic SAC activation induced by a 10-h nocodazole treatment (Endo et al., 2010). Chronic SAC activation dependent prolonged arrest in *slx5*Δ cells was reported for the first time in the present study. Over the last decades several studies revealed that *S. cerevisiae* could undergo apoptosis with characteristic markers of metazoan apoptosis (Carmona-Gutierrez et al., 2010), which makes it a suitable model organism to study apoptosis caused by antimitotics. Endo et al. previously reported that WT budding yeast cells die in mitosis with apoptosis-like features in response to a 10-h treatment with nocodazole (Endo et al., 2010). In this study, we investigated whether Slx5 is involved in the apoptosis-like response to chronic SAC activation by examining *slx5*Δ cells for apoptosis-related morphologies such as nuclear fragmentation, DNA fragmentation, and ROS production during chronic SAC activation and comparing them to those of WT cells under the same conditions. Consistent with Endo et al.’s study, we observed that prolonged mitotic arrest led to an increase in the ratio of cells displaying apoptosis-related features (fragmented nucleus, fragmented DNA, and ROS accumulation) in the WT strain. Additionally, we show the first time that the ratio of cells displaying the same apoptotic features are lower in the *slx5*Δ strain under the same conditions, suggesting that Slx5 may be necessary for the chronic SAC activation to result in apoptosis. Finally, we tested whether the role of Slx5 in the chronic SAC activation-apoptosis relation is through its ubiquitin ligase activity by candidate protein approach. We show that the role of Slx5 is not through regulating ubiquitin dependent degradation of the apoptosis-related and sumoylated candidate proteins. Although Slx5 is not required for the turnover of the apoptosis-related candidate proteins during chronic SAC activation, the involvement of Slx5 in the chronic SAC activation-apoptosis relation through its ubiquitin ligase activity cannot be completely ruled out. Slx5 could still be mediating ubiquitin dependent degradation of other apoptotic proteins that were not selected as a candidate because they were either missed in the first literature review or they hadn’t been shown to be sumoylated yet. The role of Slx5 in the chronic SAC activation-apoptosis relation may also be indirect. In other words, Slx5 may be regulating ubiquitin dependent degradation of proteins that are not indirectly involved in apoptosis. Sumo targeted ubiquitin ligases are involved in several important cellular processes including DNA damage response (Dantuma and van Attikum, 2016). Slx5 is also critical for preventing genomic instability as gross chromosomal rearrangements, such as nonreciprocal translocations and chromosome fusions, as well as increased spontaneous DNA damage are increased in *slx5*Δ cells (Zhang et al., 2006). However, recent studies suggest that sumo-targeted ubiquitin ligases can prevent or trigger genomic instability depending on the nature of the DNA damage (Nie et al., 2017), and genomic instability may induce apoptosis (Zhivotovsky and Kroemer, 2004). Although it has been demonstrated that DNA damage accumulates in cells during prolonged mitotic arrest (Hain et al., 2016), not much is known about the nature of the damage. In this respect, Slx5 may be involved in the induction of apoptosis in response to the DNA damage during the prolonged mitotic arrest, which may explain why apoptosis is decreased in *slx5*Δ cells during chronic SAC activation induced prolonged mitotic arrest. Further studies are required to elucidate the role of Slx5 in the relation between chronic SAC activation and apoptosis.

## Acknowledgment

We would like to thank Dr. Daniel J. Burke (North Carolina State University, College of Sciences, Department of Biological Sciences) for generously sending us the wild type TAP-tagged *S. cerevisiae* strains. This study was supported by the Scientific and Technological Research Council of Turkey (TÜBİTAK) with the project number: 115Z157.
